# *Subti*Wiki 2.0—an integrated database for the model organism *Bacillus subtilis*

**DOI:** 10.1093/nar/gkv1006

**Published:** 2015-10-03

**Authors:** Raphael H. Michna, Bingyao Zhu, Ulrike Mäder, Jörg Stülke

**Affiliations:** 1 Department of General Microbiology, Institute of Microbiology and Genetics, Georg-August University Göttingen, Grisebachstr. 8, D-37077 Göttingen, Germany; 2 Department of Functional Genomics, Interfaculty Institute for Genetics and Functional Genomics, University Medicine Greifswald, Jahnstr. 15a, D-17475 Greifswald, Germany

## Abstract

To understand living cells, we need knowledge of each of their parts as well as about the interactions of these parts. To gain rapid and comprehensive access to this information, annotation databases are required. Here, we present *Subti*Wiki 2.0, the integrated database for the model bacterium *Bacillus subtilis* (http://subtiwiki.uni-goettingen.de/). *Subti*Wiki provides text-based access to published information about the genes and proteins of *B. subtilis* as well as presentations of metabolic and regulatory pathways. Moreover, manually curated protein-protein interactions diagrams are linked to the protein pages. Finally, expression data are shown with respect to gene expression under 104 different conditions as well as absolute protein quantification for cytoplasmic proteins. To facilitate the mobile use of *Subti*Wiki, we have now expanded it by Apps that are available for iOS and Android devices. Importantly, the App allows to link private notes and pictures to the gene/protein pages. Today, *Subti*Wiki has become one of the most complete collections of knowledge on a living organism in one single resource.

## INTRODUCTION

In the era of low-cost and high-speed whole-genome sequencing projects, large amounts of genome data accumulate and require faithful functional annotation. These annotations are usually based on prior information that is available in public databases. A major source of such information is the knowledge that has been generated for a handful of highly studied species, i.e. the model organisms. For Gram-positive bacteria, *Bacillus subtilis* serves as the model organism. The Gram-positive bacteria include a major fraction of the biodiversity of the human microbiome, and many important pathogens such as *Staphylococcus aureus*, *Streptococcus pneumoniae*, *Clostridium difficile* or *Listeria monocytogenes*. Moreover, many species that are used in biotechnology and in diary industry such as *Bacillus licheniformis* or the lactic acid bacteria are members of this large group of bacteria. The better understanding of all these bacteria relies on our knowledge on the model organism *B. subtilis*.

Due to the developmental program of sporulation, the ease of genetic manipulation and its biotechnological importance, *B. subtilis* has attracted much interest since its original discovery by Cohn (1). This long-standing interest made *B. subtilis* one of the best-characterized organisms. With more than 2500 publications in the past two years, *B. subtilis* has continued to be in the focus of microbiological research. However, with the advance of post-genomic techniques, the type of studies has gradually shifted from genetic/ biochemical studies of individual genes or proteins to global analyses at the transcriptome and proteome levels. Moreover, *B. subtilis* has come into the focus of several projects aimed at defining the minimal genome that is required to drive an independently living cell (2–4).

Several databases that cover information on *B. subtilis* have been developed. These are either part of global efforts (BsubCyc as part of BioCyc; SubtiList, now available as part of GenoList), or specialized to specific scientific problems such as regulation or sporulation (DBTBS and SporeWeb, respectively) (5–8). To collect all available information on *B. subtilis* and to make it easily accessible to the scientific community, we have developed *Subti*Wiki, a database on all genes and proteins of *B. subtilis* (9). *Subti*Wiki is accompanied by several modules that graphically present gene expression, metabolic and regulatory pathways and protein-protein interactions (10,11). With the generation of those additional modules, *Subti*Wiki has become more and more complex. Moreover, the integration of multiple layers of different classes of information (text and figures) has become problematic due to changing gene designations. Here, we present the development of *Subti*Wiki 2.0 as a database that integrates different forms of presentation. In addition, *Subti*Wiki 2.0 provides the user with a couple of new tools that facilitate research beyond the information provided in *Subti*Wiki. Moreover, we realized the need for a mobile version of *Subti*Wiki that allows the user to easily access the most important information and to link it to private notes.

## THE *Subti*Wiki ENTRY PAGE

**Figure 1.**
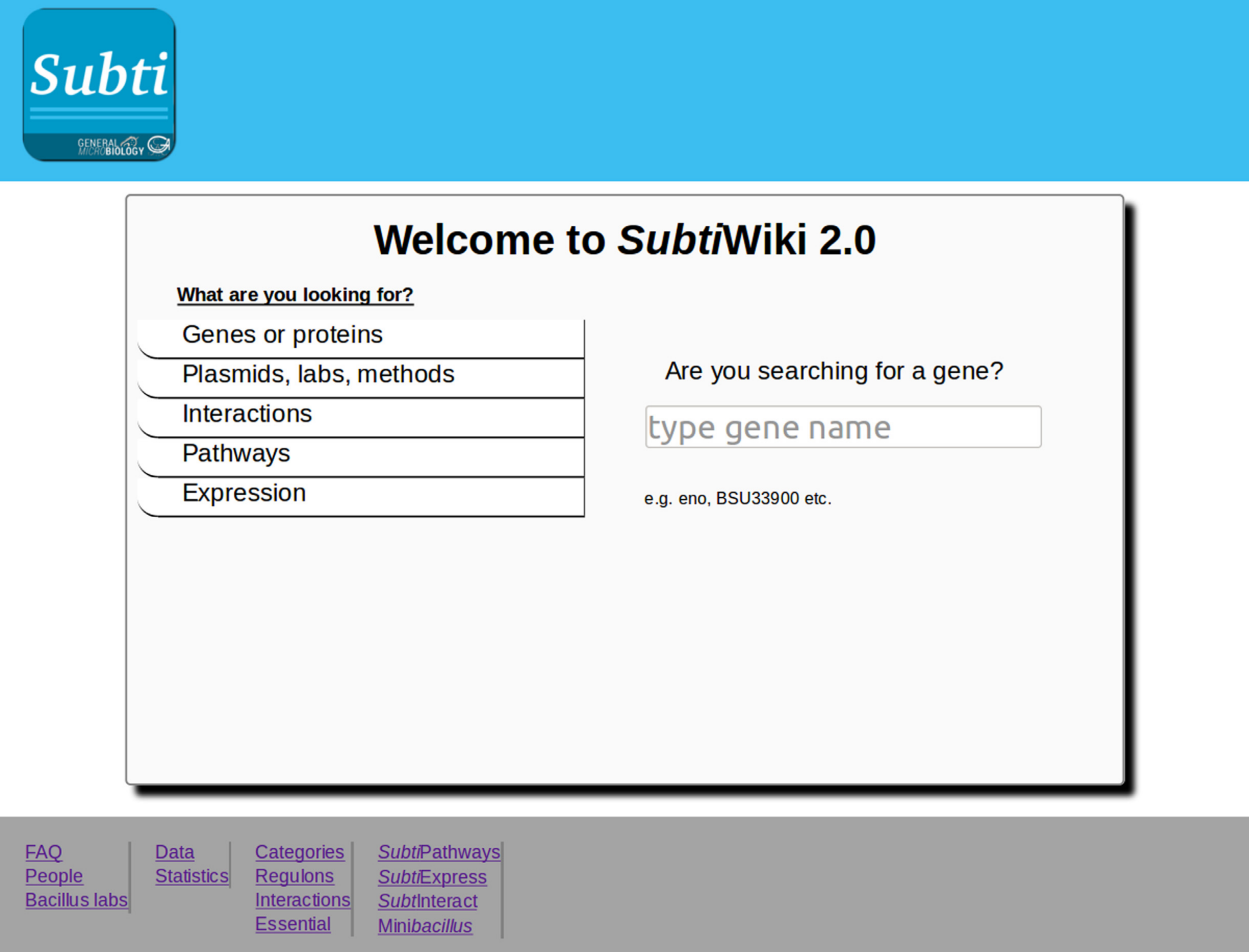
The main page of *Subti*Wiki. On the left side the user can choose the application of interest. On the right side the user simply has to type the gene/protein of interest into the search box to start the search. At the bottom of the page the user will find additional information containing statistics and data.

The common main page of *Subti*Wiki gives access to all information that is available in the database. This includes the gene or protein pages, pages for plasmids, labs and methods, as well as the pages for protein–protein interactions, metabolic and regulatory pathways, and gene/ protein expression (Figure 1). Clicking on one of the tabs gives access to a search box. A short explanation what to type in is given below the box for users that are not familiar with *Subti*Wiki. The entry page is adjusted to the respective output device (desktop, tablet or smartphone).

## THE BASIC PAGES FOR THE INDIVIDUAL GENES AND PROTEINS

**Figure 2.**
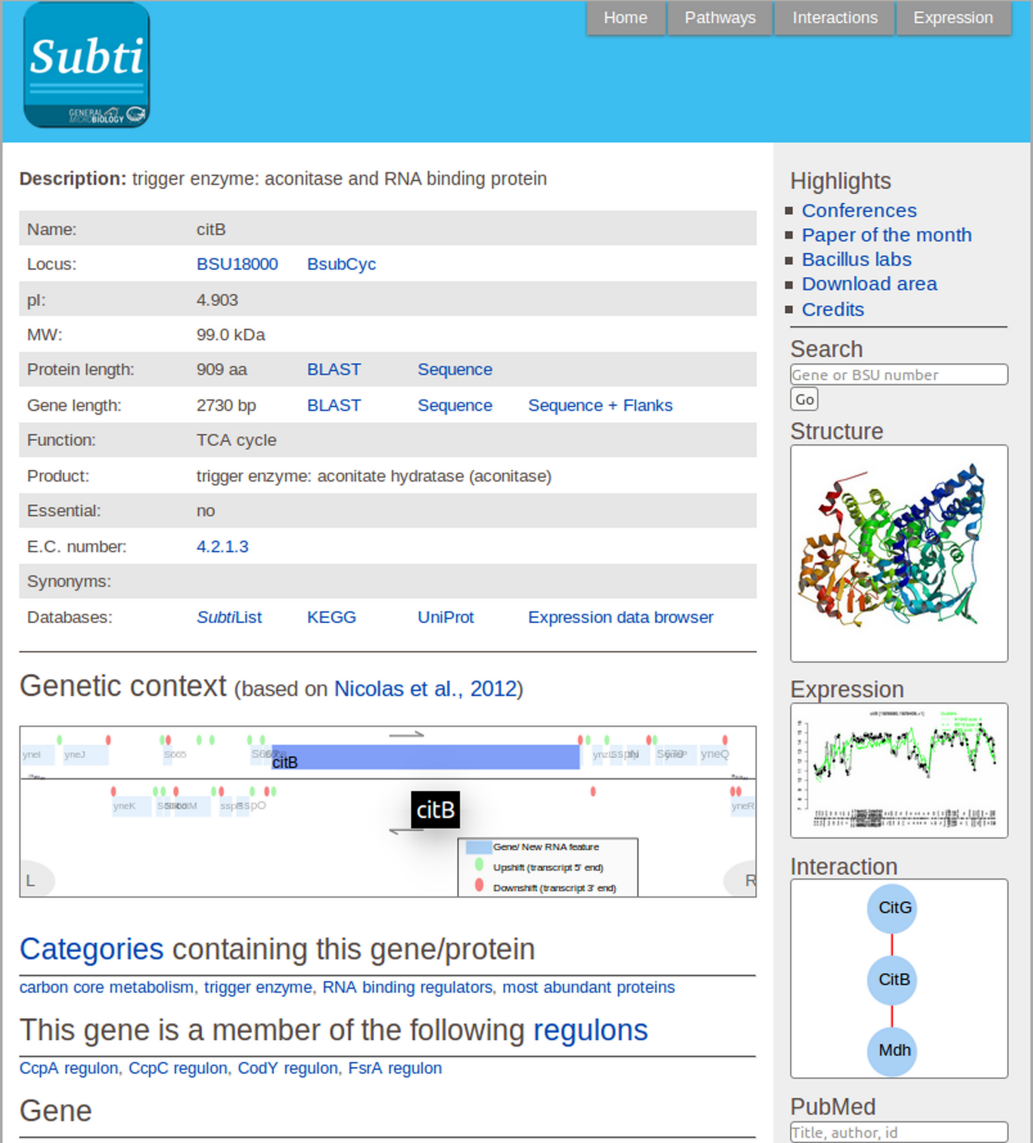
The specific page for *citB*. Each gene page shows all information for the specific gene as well as diverse links to other resources.

Since *Subti*Wiki has become very popular, we have re-designed the pages for the genes and proteins very cautiously in order to allow the users to orientate themselves quickly on the new pages (see Figure 2). In addition to all the information concerning the specific gene or protein, tabs at the very top of all pages allow direct access to the pathway, interaction, and expression pages for the concerned gene/ protein. Moreover, each page provides links to important conferences, the paper of the month, the *Bacillus* labs, and the credit page. A specific link guides the user to the download area in which all relevant files are available in Excel format. At the bottom of each page, links to *Subti*Wiki contacts, applications, and database entries are provided. The applications include direct links to Blast searches (with pre-added DNA and protein sequences), as well as to downloads of the DNA and protein sequences. The linked databases include structure (PDB, 12), enzyme classification (Expasy ENZYME, 13) and further protein resources (UniProt, 14). Moreover, other important databases of the *B. subtilis* community (SubtiList, BsubCyc and KEGG, (5,6,15)) are listed. Finally, a direct access to the *B. subtilis* expression data browser (16) is available. In all cases, the links direct the user to the pages of those databases for the specific gene or protein.

On the top of the pages, the key general information for the genes/ proteins is provided. This includes the description, the locus tag (with a link to the BsubCyc database, (5)), sizes of the gene and protein and the function (with links to download DNA and protein sequences, respectively, and to perform BLAST searches). Moreover, information on gene essentiality, the E.C. number (if any, with a link to the Expasy ENZYME database, (13)), and synonyms are listed. Finally, links to the major database entries for the gene/protein of interest are provided. Below this general information, the user finds a scheme of the genomic context of the corresponding gene. This scheme shows not only the specific gene and the adjacent genes, but gives also information on transcription directions and on transcription signals as detected in a large-scale gene expression analysis (16). Importantly, the genes and new RNA features in the picture are clickable and guide the user to the corresponding gene page. Moreover, using the L and R buttons in the diagram the user can move 10 genes up- and downstream, respectively. At the right side of each page, the user will find brief graphical presentations of the protein structure (if known), the gene expression profile under 104 conditions, and the protein-protein interactions. Clicking on the structure will bring the user to the corresponding page of the PDB database. Similarly, the expression picture gives direct access to the expression information for the gene/ protein. In the case of the interaction scheme, upon moving the mouse over a protein, a pop-up window will provide the key facts about this protein. Clicking on one of the protein names will open the corresponding *Subti*Wiki page for that protein in a new window. Finally, a searchable Pubmed box below the interaction scheme allows direct literature searches from each *Subti*Wiki page.

Below the genomic context scheme the user will find all the detailed information for the respective gene or protein. This information is presented as it was before in *Subti*Wiki, sorted with respect to functional categories, regulons, phenotypes of a mutant, detailed information on the protein (such as activity, domains, interactions, localization, etc.), information on gene expression and regulatory mechanisms, the availability of biological materials, the labs that work on the gene/protein, and references. As well established in *Subti*Wiki, all information is supported by links to the corresponding references, and links to other *Subti*Wiki pages are given whenever possible.

## THE PATHWAY MAPS

The collection of maps of metabolic and regulatory pathways consists of 49 diagrams with 1240 proteins and 201 metabolites that cover most aspects of *B. subtilis* metabolism. The pathway diagrams are accessible either from any of the gene pages or from a drop-down menu that is offered upon clicking the ‘Pathways’ button.

**Figure 3.**
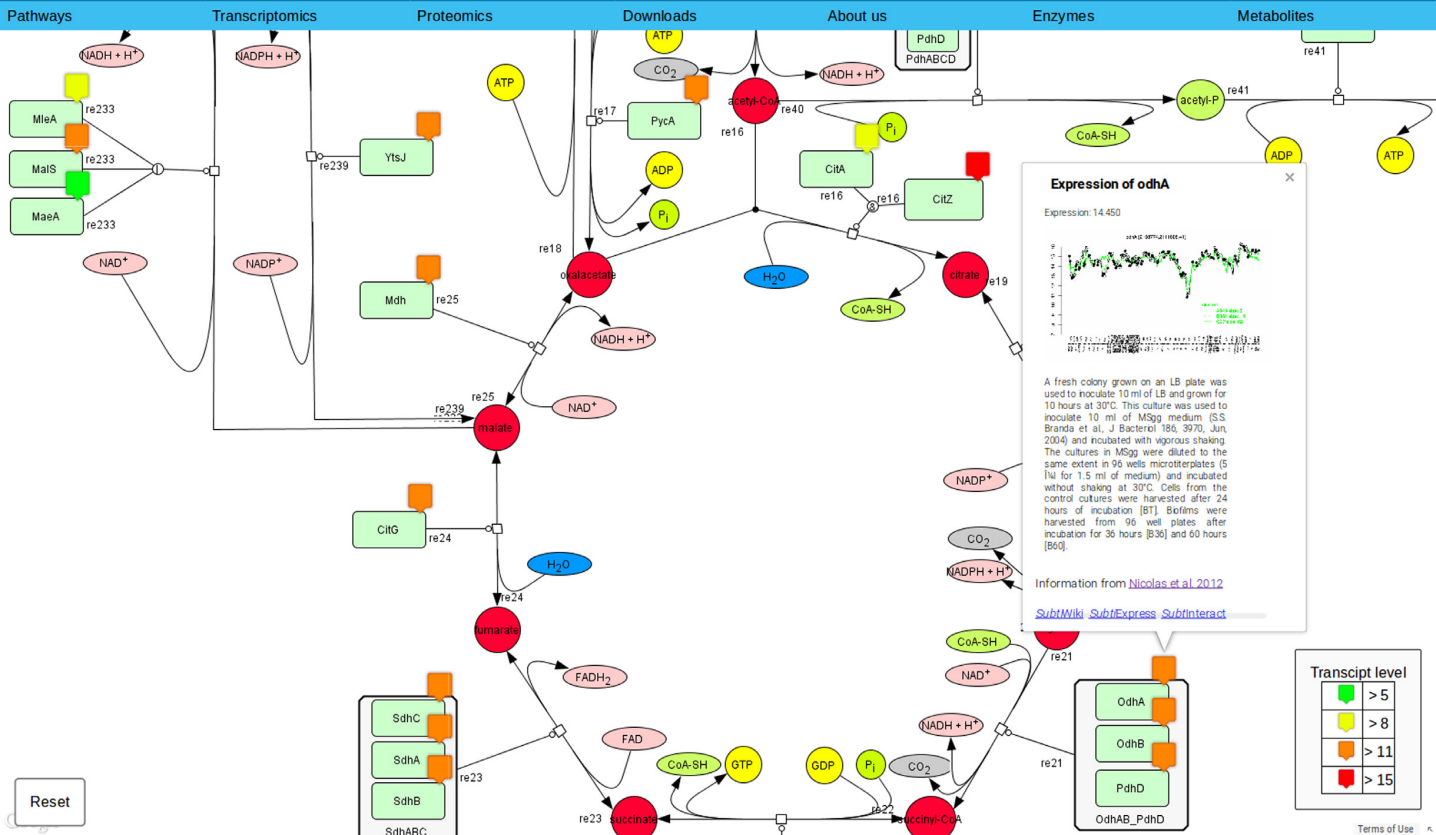
Pathway presentation in *Subti*Pathways. This application is based on the Google Maps API to generate interactive maps. On the page the user is able to project transcriptomic or proteomic data onto the maps. This results in the appearance of interactive, color-coded markers which give access to information boxes.

To enrich the pathway maps with additional information, we have added transcriptome and proteome data to correlate gene expression and protein abundances with the metabolic pathways. Moreover, this information is helpful to understand the regulatory mechanisms that are shown below the metabolic pathways. To get access to these additional sets of information, there are ‘Transcriptomics’ and ‘Proteomics’ buttons at the top of each map. Clicking on one of them opens a drop-down menu to choose a condition. The selection of one condition will add colour-coded flags to each gene/protein showing the abundance of the mRNAs or proteins (see Figure 3). The color code is explained at the bottom of the pages.

All diagrams can be downloaded as.xml files to enable the users to customize them for their specific scientific problem. Moreover, drop-down menus at the top of the maps allow the selection of any specific enzyme or metabolite in order to localize this particular molecule on the map.

All proteins and metabolites are labelled with interactive markers: in the default state, the markers give access to an information window with the basic data about the proteins. If transcriptome data are shown, the information window contains the ‘expression at a glance’ under 104 different conditions. In the ‘Proteomics’ state the information window shows the number of molecules of this protein per cell under the chosen condition (17–19). All information windows provide links to the gene-specific *Subti*Wiki, *Subti*Express and *Subt*Interact pages (see Figure 3).

## MANUALLY CURATED PROTEIN–PROTEIN INTERACTION MAPS

**Figure 4.**
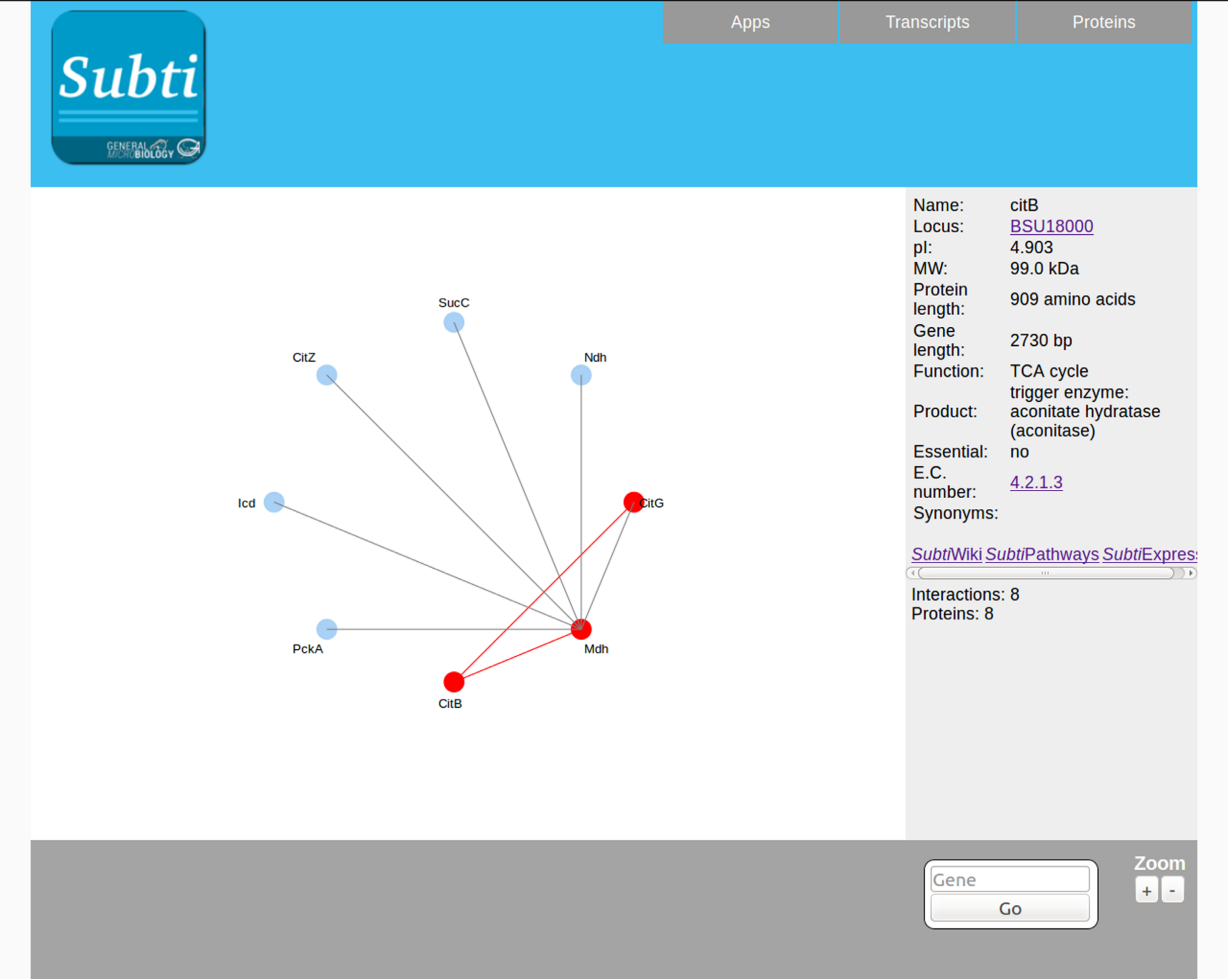
Interaction presentations in *Subt*Interact. *Subt*Interact visualizes the manually curated protein–protein interactions. The user can change the zoom level, and project transcriptomic and proteomic data on the interactive image.

A living cell is not just a sum of its components, but it requires distinct and both highly specific and dynamic protein-protein interactions. For *B. subtilis*, we have collected all interaction information from the scientific literature including several global analyses (20–23). In total, 1936 interactions with 952 participating proteins are listed. The direct interactions of each protein are shown immediately on the gene pages. Moreover, there are dedicated interaction maps that can be accessed via the button at the top of the gene pages (see above, Figure 2). These diagrams show the interaction partners of the protein of interest. Using the ‘+’ and ‘−’ button at the bottom of the diagrams, the user can change the zoom level. At level 1, the direct interaction partners of a protein are shown, whereas this extends to the partners of the partners at level 2 (and so on) (see Figure 4). To facilitate the analysis of the diagrams, the direct interaction partners are highlighted in red if the mouse goes over one protein. Moreover, at the right sight of the page, the key information for the corresponding gene/protein as well as the links to the *Subti*Wiki, *Subti*Express and *Subti*Pathways pages appear. As already explained above for the *Subti*Pathways maps, gene expression and absolute protein quantification data can be projected on the interaction maps.

## *Subti* Express PAGES PROVIDE TRANSCRIPT LEVELS, ABSOLUTE PROTEIN QUANTIFICATION AND THE TRANSCRIPTIONAL ORGANIZATION OF GENOMIC REGIONS

**Figure 5.**
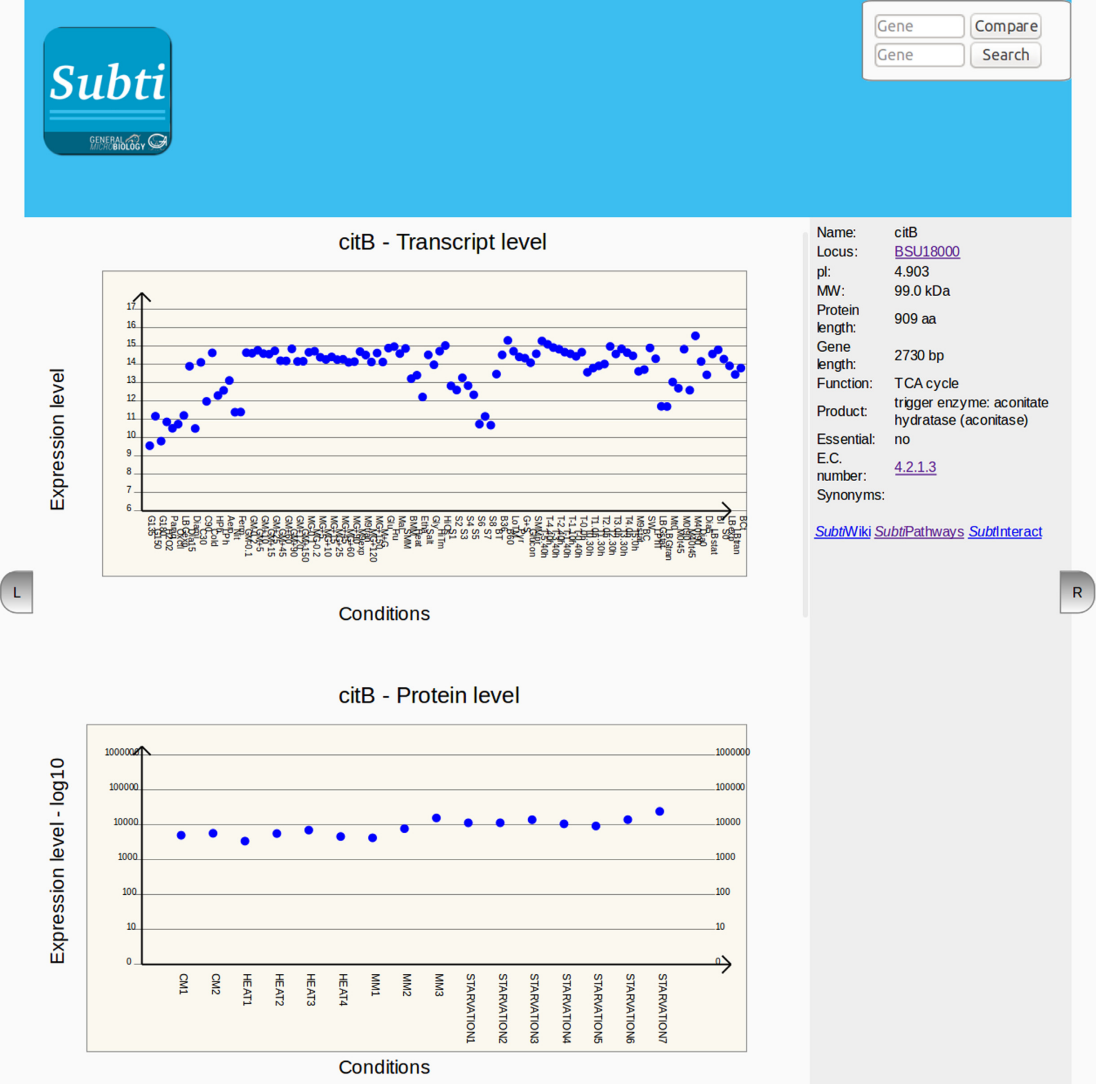
Presentation of expression data in *Subti*Express. This application provides information on gene expression levels, protein quantities as well as the transcriptional organization of the genomic region of the gene. The user can interactively explore the conditions used in the diagram and compare expression levels of the gene/protein of interest with other genes/proteins.

Expression information is provided on dedicated *Subti*Express pages. These pages are composed of three parts: The upper part shows gene expression levels under 104 different conditions, the middle part absolute protein quantities under 16 conditions, and the lower part the transcriptional organization of the respective genomic region. For the transcript and protein levels, the user can mouse over the expression dots to see information on the condition and the precise expression level (relative level for the transcriptome and protein molecules per cell for the proteome data) (see Figure 5). It should be noted that protein expression data are only available for cytoplasmic proteins. A novel feature of the *Subti*Express pages is the possibility to directly compare the expression of different genes at both the transcript and protein levels. For this purpose, the user enters the designation of the gene of interest into the ‘compare’ box at the top of the page. Expression levels of the second gene/ protein are then projected in red onto the original expression data.

## *Subti* Wiki FOR MOBILE DEVICES

With the popularity of smart mobile devices booming high in the last decade, the habits of internet users have been undergoing huge changes. Statistics have revealed that in the USA since 2013, the use of the internet from mobile devices has exceeded the use from desktop computers. In May 2014, mobile applications alone have taken up more internet usage than the combined use of browsers on desktops and mobile devices, indicating that people start to rely more on mobile apps than on browser interfaces. Thus, a mobile application for *Subti*Wiki is required to make this resource available to researchers wherever they are. Since *Subti*Wiki consists of comprehensive text which is feasible for mobile devices, we have developed the *Subti*Wiki app that is available both for iOS and for Android based devices.

The idea behind the development of the *Subti*Wiki app was that it should provide the user with the most important information ‘on the go’, but that it should also give access to the complete information in the browser version. Importantly, a second major goal in the development of the *Subti*Wiki app was the possibility to link the genes and proteins to private notes and pictures. This enhances the use of the database especially at conferences and workshops where ideas and novel pieces of information can be privately added to the genes.

The app is designed as a quick dictionary of the genes and proteins of *B. subtilis*. To find a gene, the user simply types the full or partial name of the gene or its synonym. Possible hits will then be listed, and a selection can be made. On the gene page, a brief organized summary of the gene/ protein's annotation is presented, including the function, essentiality, size of the gene and protein as well as the locus tag (see Supplementary Figure S1). The following parts of the pages provide more detailed information on functional categories, regulation, phenotypes of mutants, and biological materials that are available in the community.

**Figure 6.**
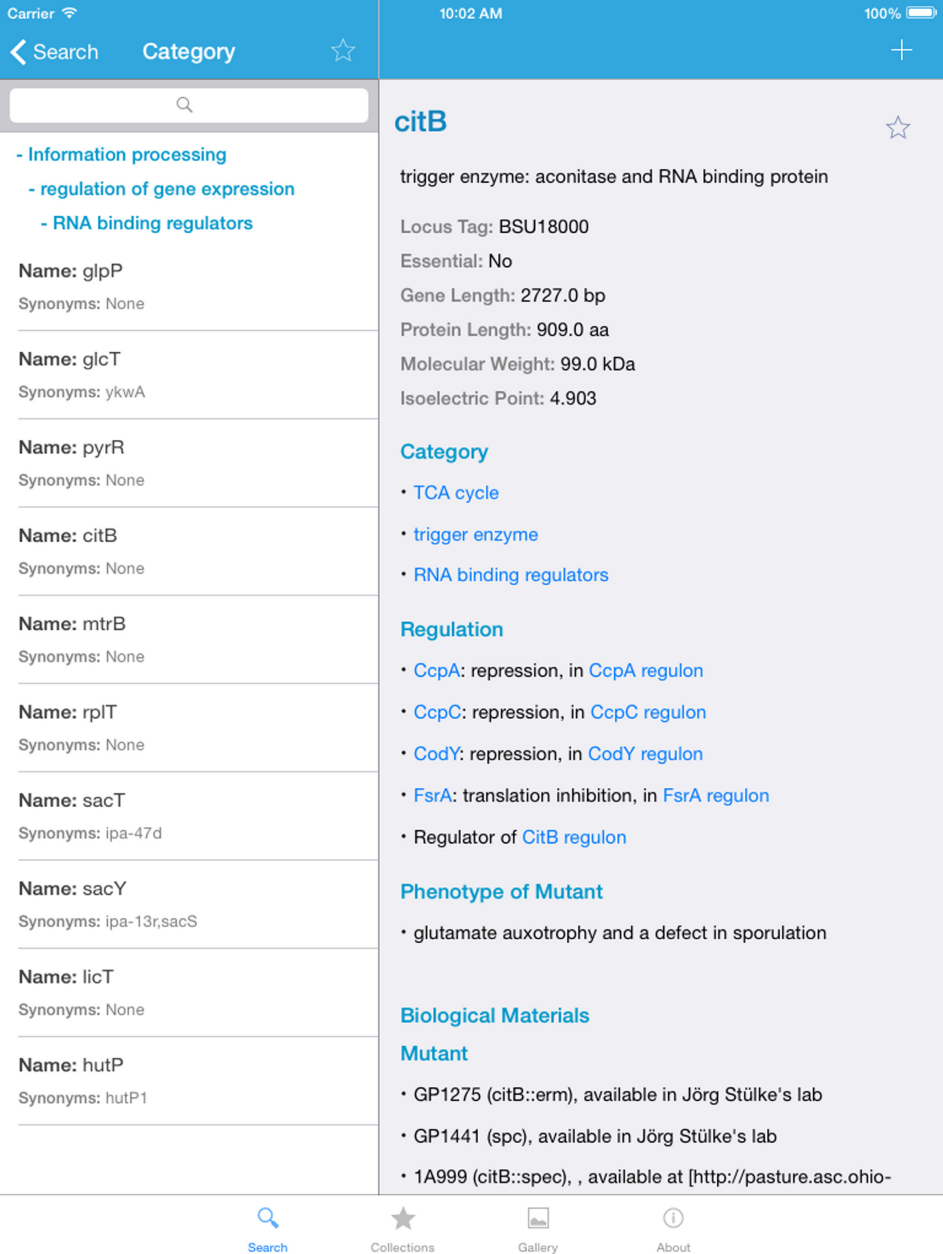
The genes in functional category ‘RNA binding regulators’. The list of all genes belonging to the functional category ‘RNA binding regulators’. All individual gene pages of the members of the category are directly accessible.

Upon clicking the underlined hyperlinks inside the gene page, the user will be redirected to either another gene page or a full list of genes belonging to a certain functional category or regulon (see Figure 6). The categories and regulation information are aimed at providing an instant overview on complete sets of functionally connected genes and proteins. Importantly, these category and regulon pages contain hyperlinked gene designations and allow the user to explore complete families of genes. This information is based on *Subti*Wiki, and all individual categories and transcription factor regulons are clickable and direct the user to pages that list all members of the respective category or regulon (see Figure 6).

On top of each gene, regulon or category page there is a star that can be clicked to mark this particular page as a favorite. This means that the information for the selected genes/ regulons/ categories will be stored on the local device, and that it is accessible even when the device is offline. Moreover, to better organize the stored data, the user can create customized lists with the ‘Collections’ tab and add the stored pages to these lists.

While the information presented in the *Subti*Wiki app is all shared with the desktop version of *Subti*Wiki, we included the possibility to add private notes as a novel feature. Researchers often go to conferences or other kinds of meetings where they are exposed to the most recent results and confidential unpublished information. To keep record of this information, private notes can be taken that will be linked to the gene page. For this purpose, the ‘+’ button at the top of the page has to be used. Then, a blank editable page appears (see Supplementary Figure S2). Here, the user can either put in a text (click ‘Done’ when finished), or a picture can be added by either taking a new photo or by selecting a photo from a gallery that is stored on the mobile device. The addition of any information will automatically add this gene page to the list of favorites, making all information available offline. The notes and pictures appear at the bottom of the gene pages (see Supplementary Figure S3), and the pictures are clickable to see them on the full screen. All the pictures that have been added are displayed in the internal gallery for a quick access and display in full screen and zoom.

Importantly, the private notes and pictures will strictly stay on the particular device on which they have been added to ensure the privacy of this information. Neither the *Subti*Wiki app nor the desktop version of the database collect any personal data of the users.

The *Subti*Wiki app can be downloaded for free from the Apple App Store and the Google Play Store for iOS and Android users, respectively.

## PERSPECTIVES

With now about 50 000 page accesses per day *Subti*Wiki has become one of the most popular databases dedicated to a single organism. It is one of the most complete inventories of knowledge on a living organism in one resource.

In the future, keeping up-to-date with the most recent scientific information will remain a key task for the development of *Subti*Wiki. In addition, we will develop database systems that further improve the internal organization of the database and that may be used for the annotation of other organisms as well.
